# Application of 3D Printing in the Surgical Planning of Trimalleolar Fracture and Doctor-Patient Communication

**DOI:** 10.1155/2016/2482086

**Published:** 2016-07-03

**Authors:** Long Yang, Xian-Wen Shang, Jian-Nan Fan, Zhi-Xu He, Jian-Ji Wang, Miao Liu, Yong Zhuang, Chuan Ye

**Affiliations:** ^1^Department of Orthopaedics, The Affiliated Hospital of Guizhou Medical University, Guiyang 550004, China; ^2^Center for Tissue Engineering and Stem Cells, Guizhou Medical University, Guiyang 550004, China; ^3^China Orthopaedic Regenerative Medicine Group (CORMed), Guiyang 550004, China

## Abstract

To evaluate the effect of 3D printing in treating trimalleolar fractures and its roles in physician-patient communication, thirty patients with trimalleolar fractures were randomly divided into the 3D printing assisted-design operation group (Group A) and the no-3D printing assisted-design group (Group B). In Group A, 3D printing was used by the surgeons to produce a prototype of the actual fracture to guide the surgical treatment. All patients underwent open reduction and internal fixation. A questionnaire was designed for doctors and patients to verify the verisimilitude and effectiveness of the 3D-printed prototype. Meanwhile, the operation time and the intraoperative blood loss were compared between the two groups. The fracture prototypes were accurately printed, and the average overall score of the verisimilitude and effectiveness of the 3D-printed prototypes was relatively high. Both the operation time and the intraoperative blood loss in Group A were less than those in Group B (*P* < 0.05). Patient satisfaction using the 3D-printed prototype and the communication score were 9.3 ± 0.6 points. A 3D-printed prototype can faithfully reflect the anatomy of the fracture site; it can effectively help the doctors plan the operation and represent an effective tool for physician-patient communication.

## 1. Introduction

Three-dimensional (3D) printing (also known as rapid prototyping technology), through layered processing and additive manufacturing, can output computerized data by “printing” the form of a solid object with complex geometry. In recent years, the rapid development of 3D printing technology combined with digital orthopedics technology has allowed its scope of applications to be extended from the industrial field to orthopedic research [1, 2]. The 3D-printed skeleton prototype has gradually overcome the two-dimensional limitation of CT and MRI data, and it three-dimensionally presents a real anatomical structure [3], moving from a virtual simulation to a realistic simulation, which can assist in accurate preoperative planning [4], as well as surgical strategy simulation, and enhance communication with patients.

A trimalleolar fracture is a type of complex ankle fracture that mostly consists of high-energy comminuted fractures. If a complete reduction is not achieved, poor alignment of the articular surface and widening or narrowing of the ankle mortise will result in traumatic arthritis and severe dysfunction. If a complete 3D replica of the fracture type can be generated before the operation, most of the details like bone fragments, medial malleolus, lateral malleolus, trochlea of astragalus, and distal tibia can be determined, which could play an important role in producing a precise strategy for the operation and will facilitate communication between doctors and patients.

In the present study, we used 3D printing technology to reconstruct the details of trimalleolar fractures in patients and evaluated its effectiveness in the surgical planning for the fracture repair and in the communication between doctors and patients.

## 2. Materials and Methods

### 2.1. General Information

The present study consisted of 30 patients, including 16 males and 14 females between the age of 31 and 42 years, with an average age of 36.5 years. The patients were classified as follows: 18 cases of traffic accident injuries, four cases of smashing injuries by heavy objects, and eight cases of sprain injuries and 16 cases of left side injury and eight cases of right side injury. Fractures were categorized according to the Lauge-Hansen classification and included 18 patients with supination-external rotation type-IV fractures and six patients with pronation-external rotation type-IV fractures. The duration between injury and the operation was 4 to 12 days, with an average of 5.4 days. The 30 patients were average randomly divided into 3D printing assisted-design group (Group A) and no-3D printing assisted-design group (Group B). Group B operation was planned and executed according to the 3D reconstruction image results from CT scan, without 3D printing fracture prototype. All patients underwent a CT scan.

### 2.2. 3D Prototype Design

Each patient was scanned by 64-slice spiral CT in the supine position. The scan settings were as follows: a voltage of 120 kV, a current of 180–220 mA, and a slice thickness of 1 mm. The scan ranged from the distal tibia and fibula area to the metatarsals, using the ankle joint space as the center to select an appropriate scan range, and included the proximal tibia and fibula. The CT scan data of the ankle joint fractures were saved as DICOM files, which were input into Mimics l0.01 software to perform region growing for threshold-based 3D image segmentation. The masked original layer was carefully patched, and each contour line was accurately selected to compute a 3D digital model of the ankle joint. The optimal quality for performing model computation was selected, and 3D imaging data of the ankle joint was obtained. The computed imaging data were saved in STL format using Mimics software and were output to a 3D printer (FlashForge Ltd., ZhengJiang, China). Polylactic acid (PLA) was used as the printing material (FlashForge Ltd., 1.75 mm in diameter); several parameters were used to section the prototype of the ankle joint fracture (see Table 1), and a 1 : 1 ratio ankle joint fracture prototype (same size as the actual fracture) was produced. Each fracture prototype total process takes approximately 5–7 hours, almost all of which is unsupervised (preprocessing = 1-2 h; printing = 4-5 h; postprocessing < 0.5 h).

### 2.3. Verification of the Validity of the 3D-Printed Prototype

Questionnaire 1 for doctors was designed according to the literature to evaluate the 3D-printed prototype [5, 6]. The questionnaire included five aspects: (1) overall satisfaction with the 3D prototype; (2) degree of verisimilitude of the 3D prototype to the actual fracture; (3) detailed demonstration of the anatomical structure of the ankle joint by the 3D prototype; (4) detailed demonstration of the fracture-fragment-displacement direction and fracture-injury degree by the 3D prototype; and (5) usefulness of the 3D prototype as a preoperative planning tool. The scores ranged from 1 to 10 points; one point indicated that the model was useless/very poor/not realistic at all, and 10 points indicated that the model was very useful/very good/very realistic.

General information about 3D printing and the basic process of printing the fracture prototype were presented to the four joint surgeons and they completed questionnaire 1 to evaluate the usefulness of the prototype of the actual fracture generated by 3D printing for preoperative planning and its verisimilitude to the actual fracture. Two surgeons designed an individualized surgical strategy according to a preoperative 3D-printed prototype of patient's fracture and a surgery simulation. Then, they performed the surgery and compared the surgical approach, the position of the internal fixation plate, with the preoperatively designed strategy. Meanwhile, the operation time and intraoperative blood loss (a tourniquet was used) of Group A and Group B were compared. The operation time was recorded from incision skin to suture, and the amount of intraoperative blood loss included the amount of blood in the gauze and the suction device. After the operation, the surgeons completed questionnaire 1 to evaluate the verisimilitude of the prototype to the actual fracture and its effectiveness for surgical planning.

Open questionnaire 2 for patients and nonmedical professionals was designed to evaluate the preoperative conversation. The questionnaire included four questions: (1) How would you rank your overall satisfaction of the conversation? (2) Is the 3D prototype useful for helping you to understand the surgical plan? (3) Is the 3D prototype useful to you to obtain a clear understanding of your condition? (4) Would you like the doctor to use a 3D prototype to communicate with you about your condition? The scores ranged from 1 to 10 points; one point indicated that the model was useless/very poor/very unrealistic, and 10 points suggested that the model was very useful/very good/very realistic.

The surgeons used the 3D-printed fracture prototype to inform the patient or the family members about the patient's conditions so that they could understand the fracture characteristics, the fracture segment location, the surgical treatment strategy, and the possible postoperative complications. After the conversation, the patients and their family members completed open questionnaire 2 to evaluate the effectiveness of the conversation and indicate their preference for communicating with the doctor using a 3D-printed fracture prototype.

### 2.4. Statistical Analysis

SPSS 17.0 was used for statistical analysis. Data are expressed as mean ± standard deviation (*X* ± *S*), and the data comparison between groups was conducted using Student's *t*-test. By correlation analysis, Pearson correlation test was used to assess the correlations between the operation time and intraoperative blood loss, *P* < 0.05 was considered as significant.

## 3. Results

**Figure 1:**
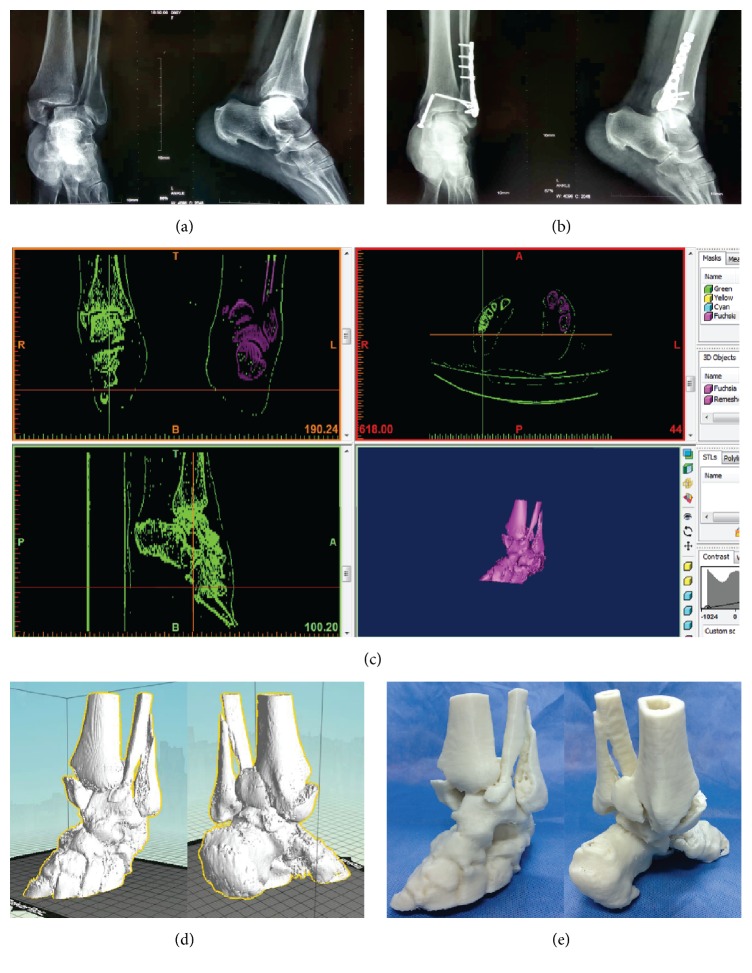
A 37-year-old female had a left trimalleolar fracture and underwent open reduction and internal fixation. 3D prototype was designed and printed by the authors; it was used for preoperative planning: (a) preoperative X-ray film; (b) postoperative X-ray film; (c) preoperative three-dimensional reconstruction by Mimics software; (d, e) digital fracture prototype and the corresponding 3D-printed 1 : 1 solid prototype.

Ankle joint fracture prototypes in Group A were successfully printed, and all 15 fracture prototypes clearly showed the anatomical structure of the ankle joint, which can help the surgeon to determine the specific type of fracture, the displacement direction of the fractured fragment, the location of bone fragments, and the injury degree of the articular surface. Through the analysis and study of the 3D-printed fracture prototypes (1 : 1 ratio), intraoperatively designed surgical plans were successfully achieved. The surgical entry point, position of the internal fixation plate, and direction and length of the implanted screws were all the same as those in the preoperative plan. The correlation between operation time and intraoperative blood loss was carried out in both Group A (*r* = 0.587, *P* = 0.022) and Group B (*r* = 0.709, *P* = 0.003), which revealed a positive correlation between the two indexes. Furthermore, the operation time and the intraoperative blood loss in Group A were less than those in Group B (Table 2), and the differences were statistically significant (*P* < 0.05). Postoperative X-ray films showed that the reduction and fixation of the ankle joint fracture were satisfactory, and the plate and the screws were in good positions. Images of a typical case are shown in Figure 1.

The results from questionnaire 1 showed that overall satisfaction and usefulness with the 3D prototype were high to the doctors (Table 3). The two surgeons who performed the surgery thought that a 3D-printed prototype could reproduce the original structure of the fracture, and it could help surgeons determine the fracture type and the displacement direction of fracture fragments, as well as evaluating the severity of the fracture through multiple angular and directional observations of the prototype, thereby producing a better overall understanding of the patient's condition. Meanwhile, an individualized preoperative strategy design and a practice operation using the prototype could shorten the operation time, lower the surgery risk, and increase the surgical accuracy and safety. The patients and family members exhibited a high degree of satisfaction regarding the use of a 3D-printed fracture prototype by the doctors to explain the details of fracture and for preoperative communication (Table 4).

## 4. Discussion

The type, size, and shape of a fracture are different in each patient, and the use of 3D printing technology can produce an individualized 1 : 1 solid prototype of the fracture. Prior to a complex fracture surgery, junior surgeons can observe the anatomical structure of the fracture using a 3D-printed prototype, and they can simulate the surgical operation to determine the size of the implants for internal fixation. The prototype can help reduce operational difficulty and shorten the learning curve [7]. Therefore, 3D printing technology can make the diagnosis as well as the surgical operation more directly visible, realistic, and specific by assisting in the clinical diagnosis, helping to plan the complex operation strategy, and allowing a simulation of the operation to be performed.

Through the analysis of the scoring results for questionnaires 1 and 2, the present study suggests that 3D printing has the following advantages: (1) A 3D-printed fracture prototype allows imaging data output to be transformed from a two-dimensional image to a three-dimensional form (from planar to three-dimensional) and from a static to a dynamic model, which gives a realistic impression to doctors. Using the 3D-prototype, the fracture can be viewed in any direction (0–360°) to allow an accurate description of fracture properties; therefore, it allows the doctor to more effectively and comprehensively understand the specific details of the patient's fracture [8, 9], verify the type of fracture, determine the movement of the fracture line and the number of fracture fragments, examine the collapse and comminuting condition of the articular surface, check for the possible presence of bone defects, and determine whether bone implantation is needed. (2) Based on a physical prototype of the actual fracture, the surgeon can design an operation strategy, analyze internal fixation methods, and choose the internal fixation type as well as the necessary exposure position. Meanwhile, surgeons can simulate the placement of the fixture on the prototype, determine the size of the internal fixation plate and screws, mark the reset point that may be used in the operation, clarify the placement position of the plate and screws, and prebend them, which can increase the accuracy of reduction and the stability of the fixation. The present study concludes that both the operation time and intraoperative blood loss in Group A were lower than those in Group B, confirming that the fracture prototype can not only help to design a scientific and rational individualized treatment strategy but also increase the surgery efficiency and reduce the operation time. (3) The use of a 3D-printed physical prototype of the actual fracture to explain the injury conditions and treatment strategy to the patient and their family members received an overall satisfaction score greater than nine points, and it helped patients and family members better understand their conditions, allowing a more effective communication. Patients and family members were satisfied with the result of this communication method, which increased patient compliance during the treatment [10].

3D printing technology can be a powerful tool for orthopedists, and it has received much attention in orthopedic applications. It has advantages for the application of surgical planning and surgery training; however, some obstacles need to be overcome. Although CT scans are made in very thin slices, the imaging modality can only provide the accumulation of the multiple slices; error can often occur between the slices. Other restrictions include system error rate and relative long learning curve. At the same time, its current applications mainly include stomatology and orthopedics. We hope that future 3D printing technology can be developed for soft tissue modeling to produce prototypes that can accurately mimic the shape and texture of human organs [11, 12], which will allow surgeons to perform simulation surgery directly on the prototype, allowing the surgeons to be more confident during operations due to sufficient preoperative practice. This technology will play an important role in increasing operation efficiency, lowering operation risk, and ensuring patient safety.

## 5. Conclusion

Our study revealed that 3D printing can reflect the anatomy of the fracture accurately; it effectively helps the doctors plan the operation and provide more effective communication between doctors and patients.

## Figures and Tables

**Table 1:** Process parameters used for 3D additive manufacturing of PLA plastic.

Parameter	Process setting for PLA
Object infill	20%
Layer height	50 *μ*m
Shell number	2
Plastic feed rate	55 mm s^−1^
Plastic filament diameter	1.65 mm
Extruder nozzle diameter	200 *μ*m
Plastic extrusion temperature	200°C
Build plate temperature	60°C

**Table 2:** Comparison of the operation time and the intraoperative blood loss of both groups.

Group	Operation time (min)	Intraoperative blood loss (mL)	Pearson correlation
Group A	71 ± 23^*∗*^	65 ± 26^*∗*^	0.587
Group B	98 ± 20	90 ± 38	0.709

^*∗*^ *P* < 0.05 versus Group B.

**Table 3:** Questionnaire for medical professionals.

Question	Subjective field	Average points
(1)	Overall satisfaction with the 3D prototype	8.8 ± 0.4
(2)	Degree of verisimilitude of the 3D prototype to the actual fracture	9.1 ± 0.5
(3)	Detailed demonstration of the anatomical structure of the ankle joint by the 3D prototype	9.3 ± 0.4
(4)	Detailed demonstration of the fracture-fragment-displacement direction and fracture-injury degree by the 3D prototype	9.2 ± 0.6
(5)	Usefulness of the 3D prototype for preoperative planning	8.9 ± 0.7

**Table 4:** Questionnaire for patients and nonmedical professionals.

Question	Subjective field	Average points
(1)	How would you rank your overall satisfaction of the conversation?	9.3 ± 0.6
(2)	Is the 3D prototype useful for helping you to understand the surgical plan?	8.7 ± 0.5
(3)	Is the 3D prototype useful for you to obtain a clear understanding of your condition?	9.4 ± 0.2
(4)	Would you like the doctor to use a 3D prototype to communicate with you about your condition?	9.3 ± 0.6

## References

[1] Qiao F., Li D., Jin Z., Hao D., Liao Y., Gong S. (2016). A novel combination of computer-assisted reduction technique and three dimensional printed patient-specific external fixator for treatment of tibial fractures. *International Orthopaedics*.

[2] Zhang W., Lian Q., Li D. (2014). Cartilage repair and subchondral bone migration using 3d printing osteochondral composites: a one-year-period study in rabbit trochlea. *BioMed Research International*.

[3] Li Z., Li Z., Xu R. (2015). Three-dimensional printing models improve understanding of spinal fracture—a randomized controlled study in china. *Scientific Reports*.

[4] Li C., Yang M., Xie Y. (2015). Application of the polystyrene model made by 3-D printing rapid prototyping technology for operation planning in revision lumbar discectomy. *Journal of Orthopaedic Science*.

[5] Schout B. M. A., Bemelmans B. L. H., Martens E. J., Scherpbier A. J. J. A., Hendrikx A. J. M. (2009). How useful and realistic is the uro trainer for training transurethral prostate and bladder tumor resection procedures?. *The Journal of Urology*.

[6] Zhang Y., Yu C.-F., Jin S.-H., Li N.-C., Na Y.-Q. (2014). Validation of a novel non-biological bench model for the training of percutaneous renal access. *The International Brazilian Journal of Urology*.

[7] Chung K. J., Huang B., Choi C. H., Park Y. W., Kim H. N. (2015). Utility of 3D printing for complex distal tibial fractures and malleolar avulsion fractures: technical tip. *Foot and Ankle International*.

[8] Dubois-Ferrière V., Assal M. (2014). Benefit of computer assisted surgery in foot and ankle surgery. *Revue Medicale Suisse*.

[9] Davidovitch R. I., Weil Y., Karia R. (2013). Intraoperative syndesmotic reduction: three-dimensional versus standard fluoroscopic imaging. *The Journal of Bone & Joint Surgery—American Volume*.

[10] Won S.-H., Lee Y.-K., Ha Y.-C., Suh Y.-S., Koo K.-H. (2013). Improving pre-operative planning for complex total hip replacement with a Rapid Prototype model enabling surgical simulation. *The Bone & Joint Journal*.

[11] Rees A., Powell L. C., Chinga-Carrasco G. (2015). 3D bioprinting of carboxymethylated-periodate oxidized nanocellulose constructs for wound dressing applications. *BioMed Research International*.

[12] Zein N. N., Hanouneh I. A., Bishop P. D. (2013). Three-dimensional print of a liver for preoperative planning in living donor liver transplantation. *Liver Transplantation*.

